# The seasonal investigation of Symbiodiniaceae in broadcast spawning, *Acropora humilis* and brooding, *Pocillopora* cf. *damicornis* corals

**DOI:** 10.7717/peerj.13114

**Published:** 2022-06-14

**Authors:** Suppakarn Jandang, Voranop Viyakarn, Yuki Yoshioka, Chuya Shinzato, Suchana Chavanich

**Affiliations:** 1 Reef Biology Research Group, Department of Marine Science, Faculty of Science, Chulalongkorn University, Bangkok, Thailand; 2 Aquatic Resources Research Institute, Chulalongkorn University, Bangkok, Thailand; 3 Atmosphere and Ocean Research Institute, The University of Tokyo, Chiba, Japan; 4 Center of Excellence for Marine Biotechnology, Department of Marine Science, Faculty of Science, Chulalongkorn University, Bangkok, Thailand

**Keywords:** Zooxanthellae, *Cladocopium*, *Durusdinium*, Next Generation Sequencing, Corals, Seasonal, Thailand, Symbiodiniaceae, Acropora, Pocillopora

## Abstract

The density and diversity of Symbiodiniaceae associated with corals can be influenced by seasonal changes . This study provided the first annual investigation of Symbiodiniaceae density and diversity associated with *Acropora humilis* and *Pocillopora* cf.* damicornis* corals in the Gulf of Thailand using both zooxanthellae cell count and next-generation sequencing (ITS-1, ITS-2 regions) techniques, respectively. The results from this study indicated that zooxanthellae cell densities in both coral species differ significantly. The number of zooxanthellae was negatively correlated with the physical environment variable (light intensity). The diversity within *A. humilis* consisted of two genera, *Cladocopium* (Cspc_C3: 56.39%, C3w: 33.62%, C93type1: 4.42% and Cspf: 3.59%) and a small amount of *Durusdinium* (D1: 1.03%) whereas *P.* cf. *damicornis* was found to be 100% associated with *Durusdinium* (D1: 95.58%, D6: 1.01% and D10: 2.7%) suggesting that each coral species may select their appropriate genus/species of Symbiodiniaceae in response to local environmental stressors. The results of this study provided some information on the coral-Symbiodiniaceae relationship between seasons, which may be applied to predict the potential adaptation of corals in localized reef environments.

## Introduction

Coral reefs act as habitats of high ecological complexity and biodiversity in the marine environment (Blackall, Wilson & van Oppen, 2015; Brown et al., 1999a). Most reef-building corals are known to sustain a symbiotic relationship with dinoflagellate algae belonging to the family Symbiodiniaceae (also known colloquially as zooxanthallae) (Eckert et al., 2020; LaJeunesse, 2002). Nutrient exchange between coral and Symbiodiniaceae underpin the success of reef ecosystems in oligotrophic waters (Ferrier-Pagès et al., 1998; Pierangelini, Thiry & Cardol, 2020). The photosynthetic products (carbon (C), nitrogen (N), and phosphorus (P)) supplied by Symbiodiniaceae are the primary means of support to host productivity, metabolism, growth and reproduction while the host coral provides the needed compounds to their symbiont for photosynthesis (Peng et al., 2012). Therefore, the symbiosis between corals and Symbiodiniaceae is essential for the development and survival of coral reefs ecosystems (Baker et al., 2013; Wilkinson et al., 2015). The density and diversity of Symbiodiniaceae associated with corals is influenced by different factors including ecological and physical conditions (Terraneo et al., 2019; Thornhill et al., 2009). Seasonal variation of the coral-Symbiodiniaceae relationship is known to effect coral health indices such as Chlorophyll *a* and zooxanthellae density, due to both physical and chemical factors of the surrounding environment (Hinrichs et al., 2013; Scheufen, Iglesias-Prieto & Enríquez, 2017). The density of zooxanthellae in several scleractinian corals around the world has been shown to be variable throughout the year, in response to seasonality (Li et al., 2008; Stimson, 1997; Zhou et al., 2017). High irradiance levels and temperatures may have severe impacts on zooxanthellae density (Fisher, Malme & Dove, 2012; Glynn, 1993; Pillay, Willis & Terashima, 2005). Most tropical reef-building corals possess their highest zooxanthellae densities during winter months, declining in the summer (Brown et al., 1999a; Fitt et al., 2001; Stimson, 1997). This has been shown to correlate with minimal coral growth rates, which occur simultaneously with low zooxanthellae density during summer season (Al-Hammady, 2013; Pillay, Willis & Terashima, 2005).

Reef-building corals possess symbiotic relationships with a genetically diverse (genera/species) range of Symbiodiniaceae, which have been attributed to coping with different environmental stressors (Rouze et al., 2019; Silverstein et al., 2011; Silverstein, Correa & Baker, 2012). However, the revised systematics of zooxanthellae were recently defined based on genetic variation (LaJeunesse et al., 2018). Seven genera of Symbiodiniaceae were described: *Symbiodinium*, *Breviolum*, *Cladocopium*, *Durusdinium*, *Effrenium*, *Fugacium* and *Greakladium* (formerly clades A, B, C, D, E, F and G, respectively). The physiological responses of corals to environmental stressors may differ among different genera/species of Symbiodiniaceae (DeSalvo et al., 2010; Rowan, 2004; Sampayo et al., 2008). The selective specific uptake of symbiont species could lead to differentiation of coral fitness and survival rates (Quigley, Willis & Bay, 2016; Tan et al., 2020). Corals associated with *Durusdinium* (formerly clade D) exhibit less damage to photosystem II (PSII) compared to others mutualistic Symbiodiniaceae genera (Jones & Berkelmans, 2011; Little, Van Oppen & Willis, 2004; Suwa, Hirose & Hidaka, 2008). Conversely, corals predominantly containing the genus *Cladocopium* (formerly clade C) had translocated large amounts of fixed carbon to their host, which could affect its capacity to remain metabolically active under high temperature conditions (Cantin et al., 2009).

To minimize the impact on coral performance, most corals do not change their Symbiodiniaceae community structure (Cunning, Silverstein & Baker, 2018; Goulet, 2006). However, the ability of corals to change symbiont proportion and/or species has consistently correlated with disturbance severity and the diversity of Symbiodiniaceae species within the host (Cunning, Silverstein & Baker, 2015; Rouze et al., 2019). Some corals adapt or acclimatize when living in more hostile environments *via* two potential adaptative mechanisms, namely, “shuffling” (change in relative abundance of Symbiodiniaceae species within a colony) and “switching” (uptake of new exogenous Symbiodiniaceae species from the environment) (Berkelmans & Van Oppen, 2006; Rouze et al., 2016; Sampayo et al., 2008). Several studies have corroborated that the genus *Durusdinium* may be found at significantly higher proportions during thermal stress than normal environmental conditions (Chen et al., 2020; Cunning, Silverstein & Baker, 2015; Dilworth et al., 2021; Poquita-Du et al., 2020; Stat et al., 2013). Moreover, the increase of opportunistic *Durusdinium* cells may help corals survive under thermal stress by assimilating and transferring the reduced amounts of organic compounds to the coral host (Fabricius et al., 2004; Ghavam Mostafavi et al., 2007; Wall et al., 2020). Consequently, bleached corals may become more resistant to higher temperatures by symbiont shuffling and/or switching (Császár et al., 2010; Davies et al., 2018). As such, the flexibility of the coral-Symbiodiniaceae relationship is important in understanding their future survival in response to environmental stressors in particular areas and coral species (Baskett, Gaines & Nisbet, 2009; Sampayo et al., 2016).

The different reproductive modes of corals can affect the establishment of the coral endosymbiosis. Generally, broadcast spawning corals acquire their Symbiodiniaceae from the surrounding environment (horizontal symbiont transmission: HT), whereas in contrast, brooding corals transfer Symbiodiniaceae cells from parents to offspring (vertical symbiont transmission: VT) (Baird et al., 2009; Sweet, 2014). In general, most hots that use horizontal symbiont acquisition are able to select a genetically diverse assemblage of symbiont partners, conferring a greater opportunity to shift their dominant Symbiodiniaceae composition, especially when corals face unstable environmental conditions (Boilard et al., 2020; Thornhill et al., 2017). Conversely, corals that use vertical symbiont transmission are theoretically able to harbor stable communities of Symbiodiniaceae due to genetic regulation of brooding corals (Quigley et al., 2018; Thornhill, Fitt & Schmidt, 2006). Therefore, Symbiodiniaceae specificity, and the stability of communities, are predicted to be improved when symbionts are transmitted vertically compared to horizontally (Baker, 2003; Thornhill, Fitt & Schmidt, 2006). However, recent studies revealed that brooding corals (VT) become more flexible by acquiring resilient species of Symbiodiniaceae from the environment (HT) and are able to transfer the new Symbiodiniaceae communities to their offspring (Quigley et al., 2018; Quigley, Willis & Kenkel, 2019).

In Thailand, almost all Acroporidae are believed to be HT species while some species of Pocilloporidae including *P.* cf. *damicornis* are VT species (Kuanui et al., 2008). Little is known about the variability and dynamics between Symbiodiniaceae and their coral hosts particularly in Thai waters. Thus, this study provides the first investigation of Symbiodiniaceae communities and diversity associated with different reproductive modes corals: *Acropora humilis* (a gonochoric broadcast spawnerr: HT) and *Pocillopora* cf. *damicornis* (a hermaphroditic brooder: VT) from the upper Gulf of Thailand over a one year period.

## Materials & Methods

### Sampling site and sample collection

**Figure 1:**
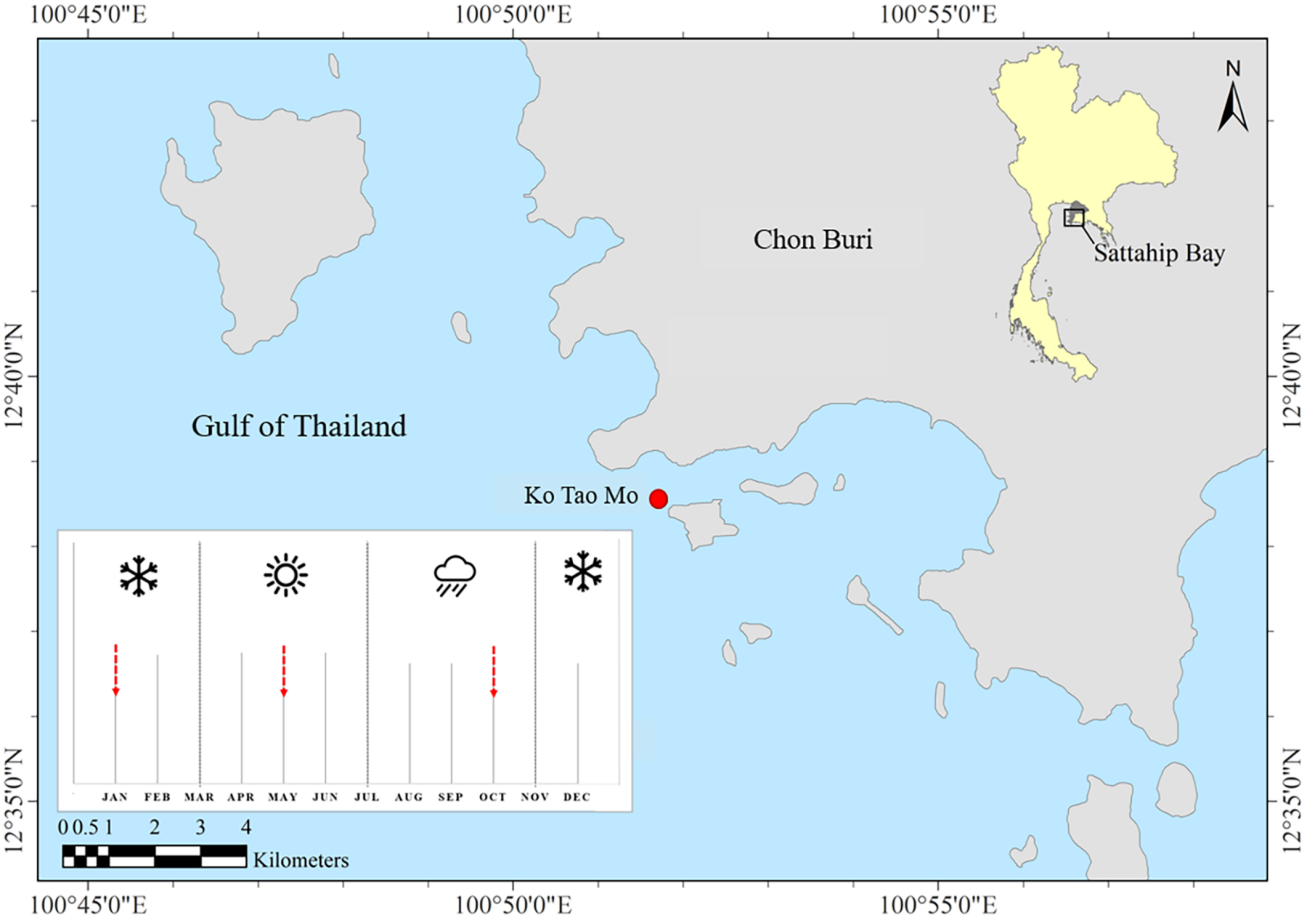
Study site and sampling period. Ko Tao Mo, Sattahip, upper Gulf of Thailand. The red arrows showed the sampling times.

Ko Tao Mo (KTM) is located at the Royal Thai Navy Base, Chon Buri province, upper Gulf of Thailand (GoT) (12°38′33.78″N 100°51′40.13″E) (Fig. 1). The average depth of the sea floor is approximately 3–15 m. Most coral communities are mainly found around the inner area of the breakwater next to KTM, after it was created in 1995. Therefore, this new coral community was estimated to be 23 years old at the time of sampling. A total of 67 species of reef-building corals were recorded. *Acropora* was found as a dominant genus in this area (Viyakarn et al., 2009). In this study, *Acropora humilis* and *Pocillopora* cf. *damicornis* corals were collected around KTM. These corals were distributed in shallow water at 5–7 m depth. Coral colonies (*n* = 15) were tagged for each species. The minimum distance between each selected colony was 3 m apart to maximize the chance of sampling distinct genetically diverse of corals. The size of tagged colonies ranged from 30–100 cm in diameter. Coral samples were collected following different seasons, referring to the local information of the Thailand Meteorological Department. These were divided into three seasons including winter (November–February), summer (March–June) and rainy (July–October). Samples were collected in January (winter), May (summer) and October (rainy) 2018, respectively. Fragments of approximately 2–3 cm from tagged colonies of each coral species and season within a year were collected by SCUBA diving. In addition, ambient sea water temperature and light intensity data were recorded using underwater temperature data loggers (Hobo, Onset Corporation LtD, Bourne, MA, USA). The average light intensity and temperature of each season were calculated based on logged data from the winter season of 2017 until the rainy season in 2018. The average temperature and light intensity (lux) was converted to PAR, µmol quanta m^−2^ s^−1^ (approximately 1 µmol m^−2^ s^−1^ = 52 lux) following the calculation method according to Bednarz et al. (2015).

### Determination of zooxanthellae density

Fresh coral fragments were collected seasonally from each of the 15 tagged colonies of *A. humilis* and *P.* cf. *damicornis* corals using hammer and chisel. Coral fragments were then transferred to laboratory and preserved in 10% buffered formalin. For zooxanthellae density determination, we adapted the decalcification method from McCowan et al. (2011). Briefly, preserved coral branches were placed in a glass container with 5% HCl solution until the coral skeleton was completely dissolved (∼4–7 days depending on size and species). The remaining tissues were rinsed and preserved in 70% ethanol. Three pieces of fixed tissue were cut into 1 × 1 cm sections and then preserved with 70% ethanol. Prepared samples were homogenized using a Nissei ACE homogenizer (Nihonseiki Kaisha LTD, Tokyo, Japan) for 5 min until the tissue was well-mixed. Subsamples (2.5 µl) of the coral tissue homogenate were immediately placed on each chamber of a Neubauer-improved bright line hemocytometer (Marien Feld, Germany) to count the number of zooxanthellae under 40x magnification. Four corner squares of each chamber were counted as one replication. To calculate the mean zooxanthellae density per cm^2^, the average total of zooxanthellae cell counted on each square was multiplied by 10^4^ ml (the volume of each chamber) and the dilution then divided by the coral tissue area (cm^2^). The average zooxanthellae density of two coral species from three different seasons were tested using a two-way ANOVA and Post Hoc tests.

### DNA extraction and PCR amplification

Small pieces of coral preserved in 95% ethanol were dried and ground using a pestle and mortar to create a coarse powder. Each powdered coral was used to extract the genomic DNA of zooxanthellae using a DNeasy Plant Mini Kit (Qiagen, Hilden, Germany) following the manufacturer’s instructions with minor modification). Crude DNA was analyzed using a NanoDrop 2000c (Thermo Fisher Scientific, Waltham, MA, USA) to examine the quality and concentration of DNA. Extracted DNA was used as a PCR template to amplify the DNA of the Symbiodiniaceae. The PCR amplifications were performed using primers for the internal transcribed spacers 1 and 2 (ITS-1 and ITS-2) genes. The targeted ITS-1 and ITS-2 regions were amplified using the specific primers for Symbiodiniaceae species identification including forward: r18Sf (5′- CGCTCTTCCGATCTCTGGAAAGTTTCATGAACCTTA T3-′) and reverse: Sym28Sr-1st (5′-TGCTCTTCCGATCTGACCTTGTRTGACTTCATGCTA -3′). PCR was carried out using AllInOneCycler™ (BIONEER, Inc., Oakland, CA, USA) with a reaction volume of 10 µl mixture for each sample which consisted of 0.2 µl PrimeSTAR GXL DNA Polymerase, 2 µl Taq buffer, 0.8 µl dNTP of Takara (TAKARA Bio Inc., Shiga, Japan), 1 µl of each forward and reverse primer, 4 µl of 0.1 µm PCR grade water (Sigma^^®^^ Life Science) and 1 µl of DNA template. The PCR conditions were 3 min at 94 °C, followed by 33 cycles of 98 °C for 10 s, 50 °C for 0.15 s, 68 for 60 s, and a final extension step of 3 m at 68 °C. PCR products were cleaned using the FavorPrep^^®^^ gel/PCR Purification Kit (FAVORGEN Biotech Corp., Wembley, WA, Australia) following the manufacturer’s protocol. Each purified sample was then used for the second PCR. The unique short nucleotides barcode (Eurofins Scientific, Luxembourg, Germany) including forward and reverse were performed. Here 90 pairs of different primers (90 coral samples) were used to include in the second PCR mixture. Each 10 µl PCR sample included the following components: 0.24 µl PrimeSTAR GXL DNA Polymerase, 2.4 µl Taq buffer, 0.96 µl dNTP of Takara (TAKARA Bio Inc., Shiga, Japan), 1 µl (0.1 µM) of each unique primer combination of forward and reverse primers, 3.4 µl Sigma water and 1 µl of purified PCR product. The PCR conditions were 3 min at 94 °C, followed by 13 cycles of 98 °C 10 s, 68 °C 60 s. Second PCR products were run in a 1.5% agarose gel with EcoDye Nucleic Acid Staining Solution (BIOFACT Co., Ltd., Daejeon, Korea) to check the targeted size of DNA.

### Next-generation sequencing and data analysis

Each successful PCR amplicon was pooled together in an equimolar concentration for sequencing. Pooled samples were cleaned using SPRI Based Size Selection (Beckman Coulter, Inc, USA). DNA libraries then were quantified on Agilent Bioanalyzer 2100 (Agilent Technologies, Palo Alto, CA, United States) and qPCR (Thermo Fisher Scientific, Waltham, MA, USA) to examine the concentration and average DNA fragment size (bp). We used 4nM of DNA libraries and made a dilution to 8 pM followed by sequencing on an Illumina Mi-Seq platform (Illumina, San Diego, CA, United States) by the Atmosphere and Ocean Research Institute, The University of Tokyo, Japan. The analysis pipeline and analytical procedures were carried out following Shinzato et al. (2018). The raw data were submitted to the NCBI Sequence Read Archive (BioProject ID: PRJNA747703). For the statistical analysis of difference in Symbiodiniaceae diversity, Shannon and Simpson indices were calculated to assess the level of alpha diversity in both corals (Simpson, 1949). The Kruskal–Wallis test was performed to test the difference in the Symbiodiniaceae community during different seasons. A permutation multivariate analysis of variance (PERMANOVA) test was used to compare the Symbiodiniaceae community compositions among coral species. To visualize the beta diversity of Symbiodiniaceae community structure among coral hosts, non-metric multidimension scaling (nMDS) based on Bray-Curtis dissimilarity was plotted using Phyloseq and VEGAN packages in R software (McMurdie & Holmes, 2013; Oksanen et al., 2020).

## Results

### Local environment condition

**Figure 2:**
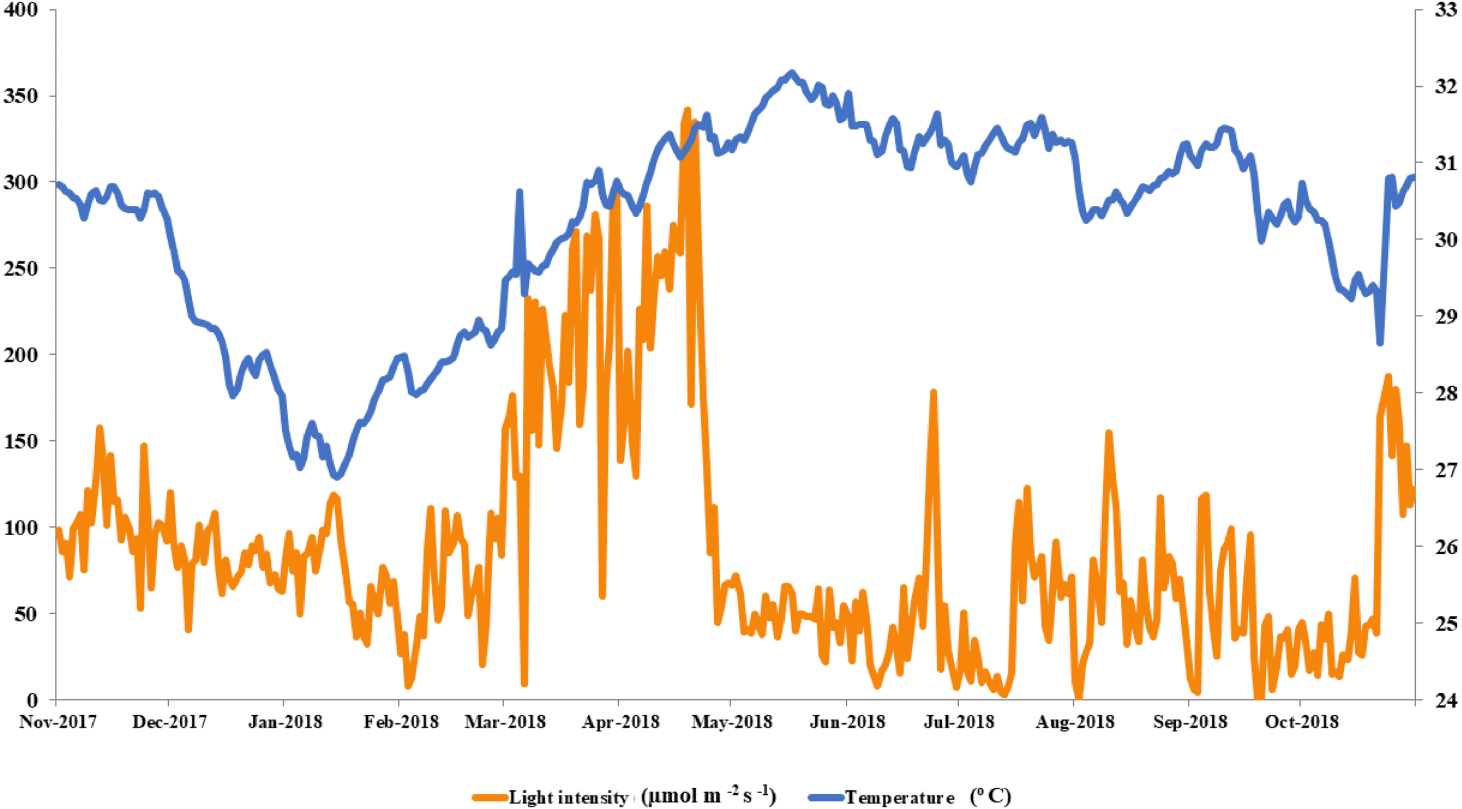
Average light intensity and temperature from winter to rainy seasons at Ko Tao Mo, Chon Buri, Upper Gulf of Thailand during November 2017 to October 2018.

The local conditions at Ko Tao Mo reef were continuously recorded during each season over a year. The seasonal minimum and maximum temperatures were recorded in January (26.90 ^o^C) and May (32.18 °C) contrasting with light intensity, which was lowest in August and September (1 µmol m^−2^ s^−1^) and highest in April (342 µmol m^−2^ s^−1^), respectively. An average local light intensity and temperature in each season was found to be 81.25 ± 2.50, 121.86 ± 8.49, 55.90 ± 3.84 µmol m^−2^ s^−1^ and 28.80 ± 0.11, 31.06 ± 0.06 and 30.67 ± 0.06 °C following winter (November–February), summer (March–June) and the rainy season (July–October), respectively (Fig. 2). While both light intensity and temperature were highest in the summer, field observations found no sign of coral bleaching (though a slightly paler color was recorded in both *A. humilis* and *P.* cf. *damicornis* colonies) during 2018.

### Zooxanthellae density

**Figure 3:**
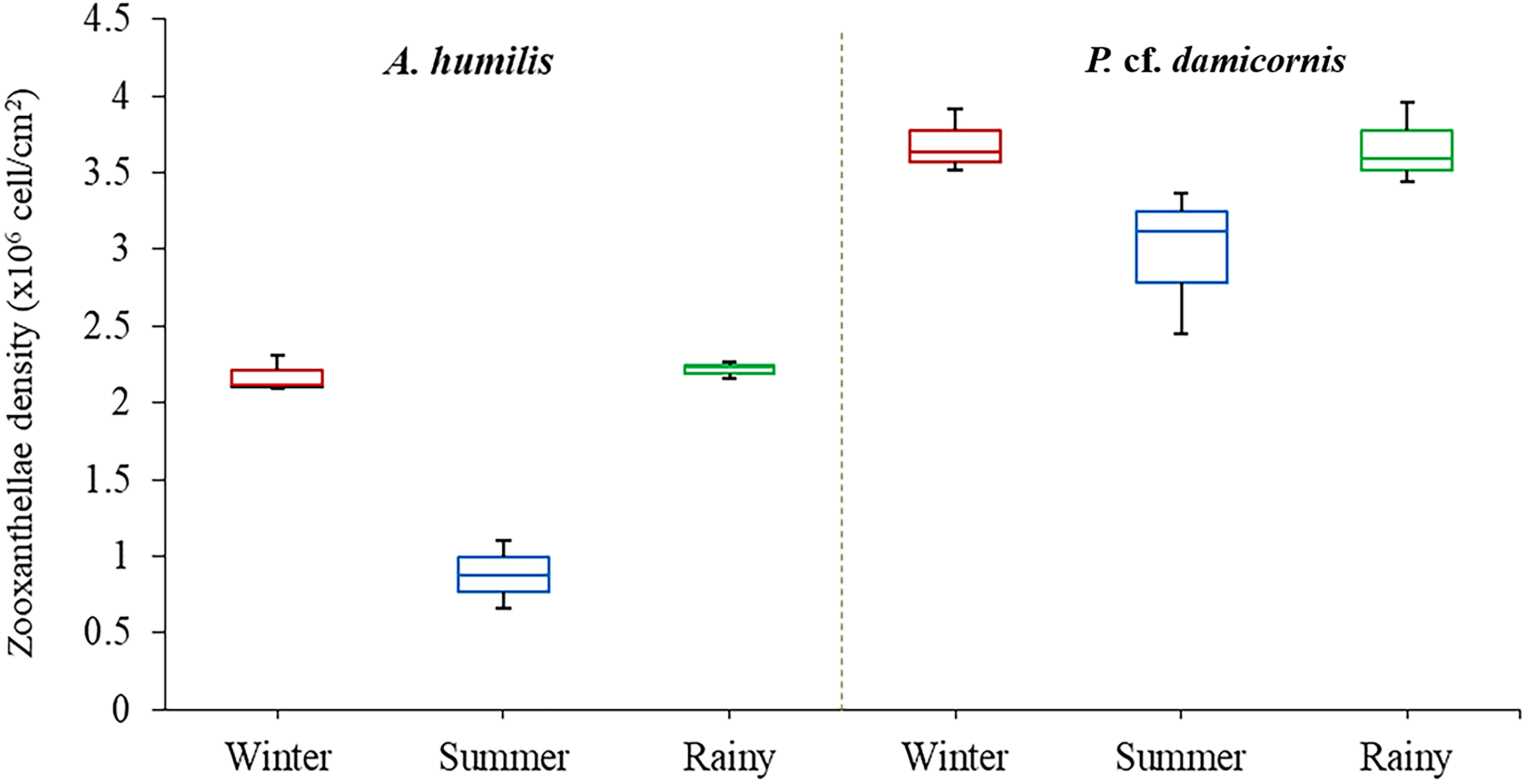
Box plots describing average of zooxanthellae densities of *A. humilis* and *P.* cf. *damicornis* during the winter, summer and rainy seasons. The median abundances and the interquartile ranges have been indicated in the plots.

Average zooxanthellae density (ZDs) in *A. humilis* and *P.* cf. *damicornis* across the three seasons ranged from 0.66 × 10^6^ to 3.69 × 10^6^ cells/cm^2^. The mean ZDs in *P.* cf. *damicornis* were twice as high (3.45 ± 0.62 × 10^6^ cells/cm^2^) as *A. humilis* (1.75 ± 0.42 × 10^6^ cells/cm^2^) (Supplementary 2). The variation of physical factors in different seasons was found to significantly impact ZDs especially in *A. humilis*. Pearson correlation among zooxanthellae cell density, light intensity and temperature in this study is presented in Table 1. The density of zooxanthellae cells showed strong negative correlations with light intensity and a weak inverse correlation between temperature. The mean ZDs in both species showed the lowest number during the summer with *A. humilis* having 0.8 ± 0.18 × 10^6^ cells/cm^2^ and *P.* cf. *damicornis* having 3.66 ± 0.42 × 10^6^ cells/cm^2^. The highest ZDs in *P.* cf. *damicornis* were found to occur in winter (3.69 × 10^6^ cells/cm^2^) while the highest in *A. humilis* was in the rainy season (2.21 ± 0.03 × 10^6^ cells/cm^2^) (Fig. 3). However, the comparison of ZDs between winter and rainy season in both corals was not found to be significantly different (*p* > 0.05) (Supplementary 3).

**Table 1:** Summary of two-way ANOVAs analyses on Symbiodiniaceae density in *A. humilis* and *P.* cf. *damicornis* at different local light intensities and temperature.

	Light intensity	Temperature	*A. humilis*	*P. damicornis*
	(µmol m^−2^ s^−1^)	(°C)	(×10^6^ cells/cm^2^)	
Light intensity	1			
Temperature	.610	1		
ZDs of *A. humilis* (×10^6^ cells/cm^2^)	−0.98 ^**^	−0.64	1	
ZDs of *P.damicornis* (×10^6^ cells/cm^2^)	−0.76^*^	−0.53	0.71	1

**Notes.**

** Correlation is significant at the 0.01 level.

* Correlation is significant at the 0.05 level.

### Diversity and composition of Symbiodiniaceae of *A. humilis* and *P.* cf. *damicornis* corals

**Figure 4:**
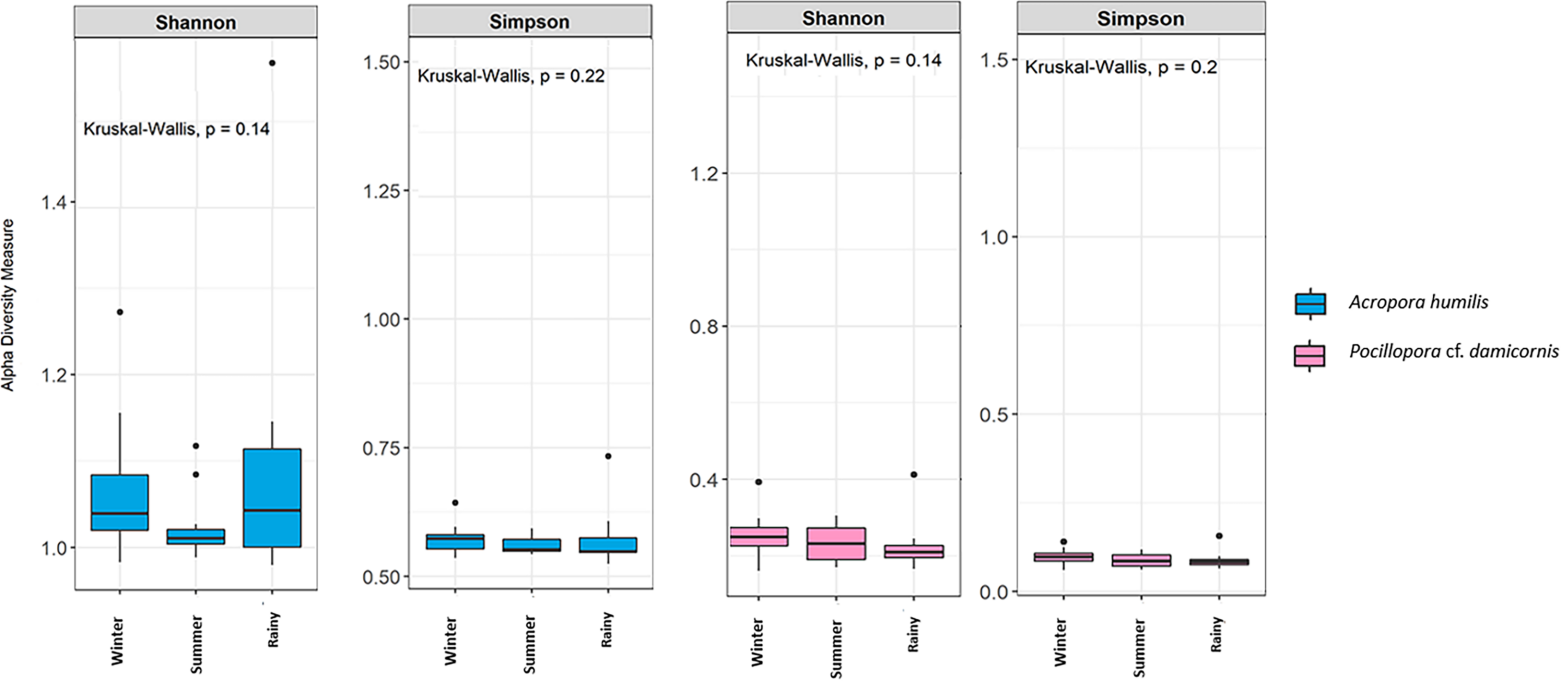
Box plots showing alpha diversity indices of Symbiodiniaceae community from two coral species, *Acropora humilis* (*n* = 15) and *Pocillopora* cf. *damicornis* (*n* = 15) in different seasons. Shannon and Simpson indexes reflects the evenness and diversity of Symbiodiniaceae species, respectively. Median values and interquartile ranges were indicated in the plots. Filled circles represent outliers. Differences were assessed by non-parametric Kruskal–Wallis test, and *p*-values are shown in each box (*p* > 0.05).

**Figure 5:**
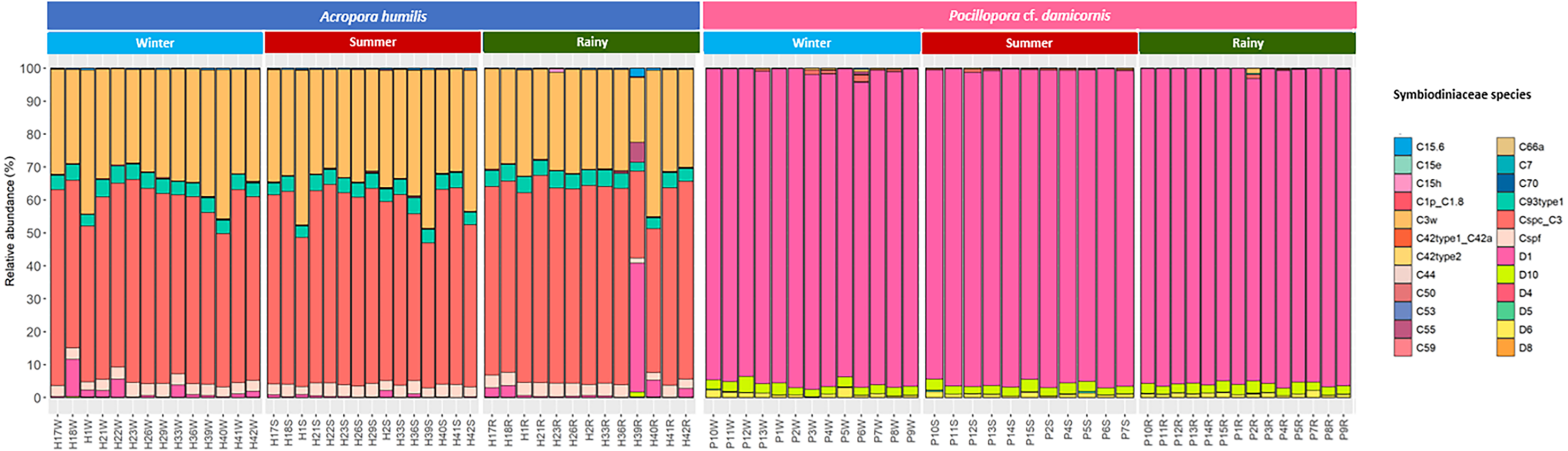
Relative abundance of Symbiodiniaceae communities in *A. humilis* and *P.* cf. *damicornis* across different seasons in 2018.

**Figure 6:**
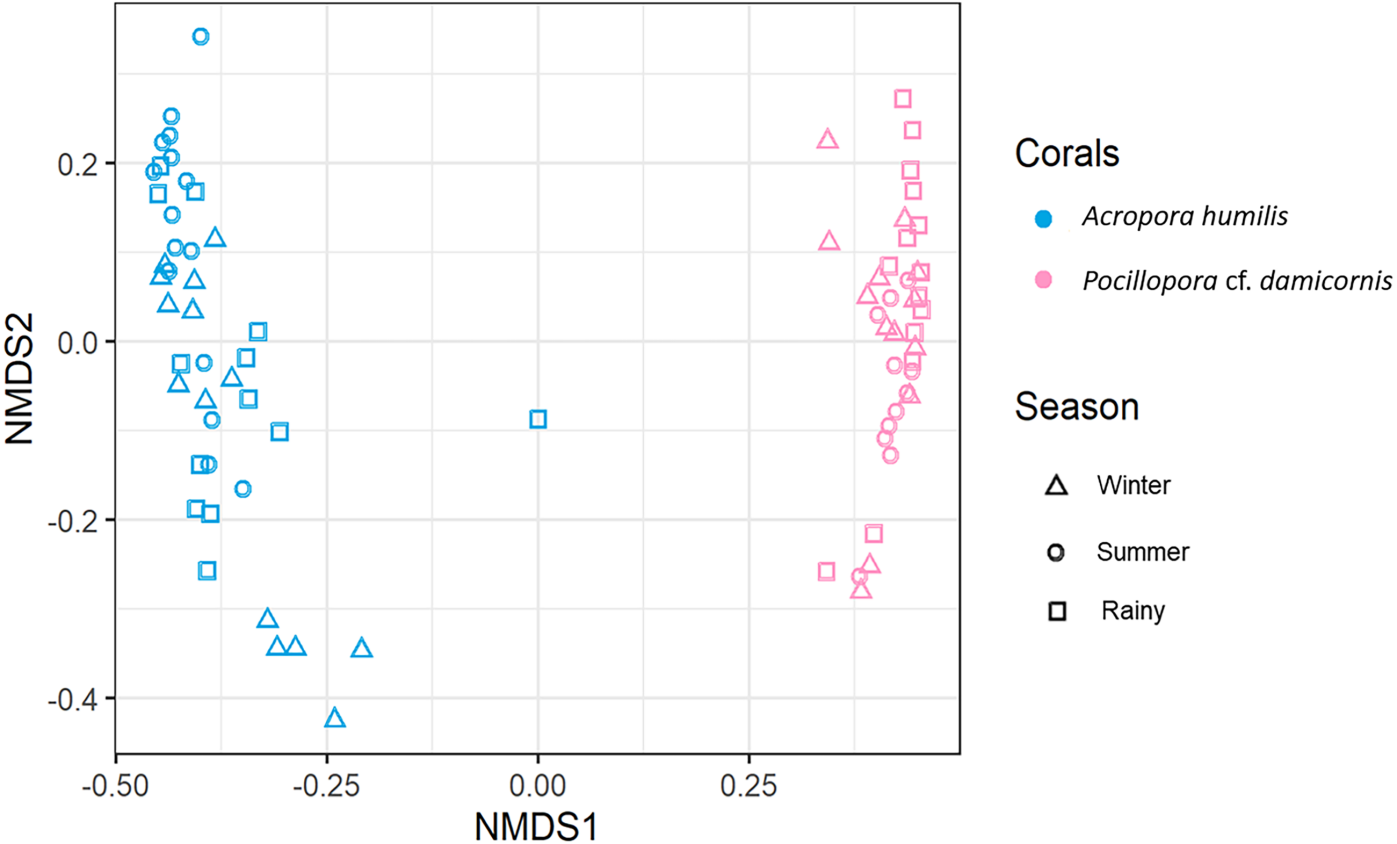
Non-metric multidimensional scaling (nMDS) plotting of the Symbiodiniaceae species composition of two coral species based on of Bray-Curtis dissimilarity indices.

Genomic DNA was obtained from 80 colonies of two coral species from three seasons. Sequencing of the ITS-1 and ITS-2 amplicons yielded 3,450,698 raw demultiplexed reads. Adaptor and quality trimmed reads resulted in 1,488,632 raw contigs and yielded 1,137,320 contigs after chimera removed reads from 80 individuals. An average 14,217 contigs/sample (Supplementary 4). Based on the GeoSymbio database, two genera of Symbiodiniaceae were detected: *Cladocopium* (14 species) and *Durusdinium* (six species). The alpha diversity indices (Shannon and Simpson) of coral showed that *A. humilis* appeared to associate with Symbiodiniaceae communities with higher levels of diversity than *P.* cf. *damicornis* (Fig. 4) (Supplementary 4). A total of 14 species were identified in the two coral species, with *A. humilis* consisting of two dominant species including Cspc_C3 (56.39%), C3w (33.62%) and other minor species C93type1 (4.42%), Cspf (3.59%) D1 (1.03%) and other species (0.95%). On the other hand, the majority of *Durusdinium* was found in *P.* cf. *damicornis* following D1 (95.58%), D6 (1.01%) D10 (2.70%) and other species (0.71%) (Fig. 5). A Bray-Curtis based nMDS plot illustrated a significant difference in Symbiodiniaceae communities between *A. humilis* and *P.* cf. *damicornis* (PERMANOVA, *p* < 0.001). The number of valid alignments of Symbiodiniaceae species across the different seasons remained relatively consistent in *A. humilis* and *P.* cf. *damicornis* throughout the year but a greater dominance of *Durusdinium* D1 was found in one colony of *A. humilis* during the rainy season (Fig. 6).

## Discussion

Our study confirmed that each coral genus contained significantly different numbers of zooxanthellae. However, the average zooxanthellae densities in both *A. humilis* and *P.* cf. *damicornis* (1.75 and 3.44 × 10 ^6^cells/cm^2^) were within the range shown in other studies in the Pacific and Indian ocean with approximately 1–3 × 10^6^ cells/cm^2^ (Fitt et al., 2001; Li et al., 2008; Pillay, Willis & Terashima, 2005; Qin et al., 2019; Stimson, 1997; Xu et al., 2017; Zhou et al., 2017). Several factors can apparently determine the number of zooxanthellae cells in each coral species (Fitt et al., 2009; Yuyama et al., 2016). One such characteristic is tissue thickness, which is correlated with the capacity to absorb light (A_max_) by each coral (Scheufen, Iglesias-Prieto & Enríquez, 2017). For example, thinner tissue layers and smaller polyps such as are found in *Acropora* contained lower numbers of zooxanthellae than corals from genera such as *Porites* and *Stylophora* which has a thicker tissue, such that the thicker tissue corals require a higher symbiont densities or pigmentation to reach A_max_ (Ben-Zvi, Eyal & Loya, 2015; Li et al., 2008; Loya et al., 2001; Scheufen, Iglesias-Prieto & Enríquez, 2017; Tambutté et al., 2007). Moreover, corals harbored different species of Symbiodiniaceae, which perform differently in term of photosynthetic activity (Cantin et al., 2009; Yuyama et al., 2018). Generally, *Durusdinium* has a relatively lower potential to provide photosynthetically fixed carbon to the coral host compared with *Cladocopium* (Sproles et al., 2020). Other studies have confirmed that the same coral species which contained different genera of Symbiodiniaceae show a lower concentration of photopigments per zooxanthellae cell in *Durusdinium*-colonies, resulting in a higher number of zooxanthellae cells required by those corals (Wall et al., 2020; Yuyama & Higuchi, 2014). This may explain the different symbiont densities detected in *A. humilis* (thin tissue) and *P.* cf. *damicornis* (thick tissue) in this study. It is however important to state that the variation of zooxanthellae density also depends on sampling depth, coral species, local environmental factors (Al-Hammady, 2013; Brown et al., 1999b; Ladrière et al., 2014; Stimson, 1997; Xu et al., 2017), zooxanthellae count preparation method (McCowan et al., 2011) and interval time of sample collections.

Seasonal changes have been shown to influence zooxanthellae density variation in both corals (*A. humilis* and *P.* cf. *damicornis*), especially because the high light intensity and temperature during summer are negatively correlated with zooxanthellae densities (Hill & Ralph, 2007; Pillay, Willis & Terashima, 2005). Rising temperature and light intensity were found to cause a reduction in zooxanthellae cell counts in several shallow water corals in Thailand (Andaman Sea) (Brown et al., 1999b). Likewise, assessments of zooxanthellae function have also been carried out *ex-situ* based-on colonies found near the present study site of Ko Tao Mo. The corals, *A. millepora* and *Platygyra sinensis*, were shown to have a statistically significant reduction in photosynthetic rate (*Fv/Fm*) under higher light intensities (Kuanui et al., 2020; Kuanui et al., 2015). Thermal stress during coral exposure to high UV radiation and elevated temperature resulted in an overproduction of reactive oxygen species (ROS) by intracellular endosymbiont cells (Nielsen, Petrou & Gates, 2018; Vidal-Dupiol et al., 2009). The enhanced production of ROS and antioxidant expression in corals not only causes irreversible DNA damage but also has the consequence of increased metabolic activity, which becomes toxic to the host (Downs et al., 2002; Wang et al., 2017). Therefore, scleractinian corals respond to their physiological stress by expelling the endosymbiont cells to minimize intracellular damage and balancing of energy dissipation efficiency during environmental stress (Kemp et al., 2014; Nielsen, Petrou & Gates, 2018; Weis, 2008). On the other hand, corals established higher zooxanthellae density during winter in some subtropical and tropical reef regions (Costa, Sassi & Amaral, 2004; Ferrier-Pagès et al., 2011; Fitt et al., 2000; Pillay, Willis & Terashima, 2005; Stimson, 1997), which may be linked to an optimum range of light and temperature for photosynthetic activity in coral at this particular site (Kleypas, McManus & MeÑEz, 1999; Schoepf et al., 2015). Pillay, Willis & Terashima (2005) also reported that the higher zooxanthellae densities were recorded during winter, which may be due to the physiological seasonal adaptations of corals during low light conditions for harvesting capacity.

The Symbiodiniaceae community in the present study was similar to previous studies in Thailand, with only two genera, *Cladocopium* and *Durusdinium,* being found both in the Gulf of Thailand and Andaman Sea but different coral genera were identified (Chankong et al., 2020; Pootakham et al., 2018). The community structure of Symbiodiniaceae with particular coral species also showed a similar pattern to those in Xinyi and Palau reefs, in which, *Acropora* spp. hosted *Cladocopium* while *Pocillopora* spp. contained *Durusdinium* D1 almost exclusively (Qin et al., 2019; Wham, Ning & LaJeunesse, 2017). Nevertheless, the different patterns of the same coral genus associated with Symbiodiniaceae genus/species is known to occur across both local scales and wide geographic zones (Rodriguez-Lanetty et al., 2001; Ulstrup & Van Oppen, 2003; Van Oppen et al., 2005). Several studies in the Great Barrier Reef (Lizard Island) were found to show the opposite trend of Symbiodiniaceae community in Acroporidae and Pocilloporidae, with *Acropora* being associated several species of Symbiodiniaceae, such as *Symbiodinium* (A3_302), *Cladocopium* (C1_769, C42.2_1367, and C3nn_1031) while *Pocillopora* associated with almost 100% of *Cladocopium* (C1_769) (Ziegler et al., 2018).

This study indicates that corals showed a strong specificity to a particular Symbiodiniaceae genus which may correlate with the pattern of symbiont acquisition (Fabina et al., 2012; LaJeunesse et al., 2004; Stat et al., 2008). The coral-Symbiodiniaceae association may be influenced by several factor including environmental variability, physiology, life history, and reproductive background (LaJeunesse et al., 2004; LaJeunesse et al., 2010). Approximately 80–90% of reef building corals in the Indo-Pacific region are broadcast spawners, which produce zooxanthellae-free gametes (Richmond & Hunter, 1990; Yuyama et al., 2016). Generally, *Acropora* spp. are considered to horizontally transmit symbionts and can harbor several species of Symbiodiniaceae to cope with environmental stress, such as was found for *A. humilis* in this study (Gong et al., 2019; LaJeunesse et al., 2010; Putnam et al., 2012; Quigley, Willis & Bay, 2017). The offspring of these hosts probably reflected local environmental conditions during the early life stages when the symbiosis with Symbiodiniaceae was initially developed (LaJeunesse et al., 2004). Free-living Symbiodiniaceae cells, both in the water column and sediment, are an important natural pool for corals during larval or primary polyp stage and/or under critical conditions (Adams, Cumbo & Takabayashi, 2009; Hoegh-Guldberg et al., 2008). Work by Yamashita et al. (2014) found that *Cladocopium* were detected mostly in the sediment while *Symbiodinium* and *Durusdinium* were found in the water column. This could explain how Symbiodiniaceae cells in the sediment play a significant role in symbiont uptake in *A. humilis.* On the other hand, *Pocillopora* has been shown to associate with *Durusdinium*. *Pocillopora* cf. *damicornis* in Thailand has been reported as a brooding coral, which releases planulae containing Symbiodiniaceae cells (Kuanui et al., 2008). The Symbiodiniaceae community of these corals’ offspring are hypothesized to be more similar to their mother colony due to the vertical symbiont transmission process (Hirose & Hidaka, 2006; Quigley et al., 2018). Consequently, most VT corals including *P.* cf. *damicornis* possess greater stability with Symbiodiniaceae species across biogeographic gradients including Thai waters with almost 100% association with *Durusdinium* D1 (Chankong et al., 2020; Muller-Parker & Davy, 2001; Pootakham et al., 2021; Thornhill, Fitt & Schmidt, 2006). However, there is evidence that some brooding corals exhibit a mixed-mode acquisition of Symbiodiniaceae (vertical transmission and horizontal symbiont acquisition). Consequently, these corals may become more tolerant to stressful conditions than previously thought by up-taking the resilient Symbiodiniaceae species from the environment (Epstein et al., 2019; Quigley et al., 2018; Quigley, Willis & Kenkel, 2019).

*Acropora humilis* in this trial hosted *Cladocopium* species and were found to be more susceptible to bleaching when higher light intensity was presented. Similarly, several studies have confirmed that *Acropora* spp. tend to be more susceptible to bleaching, which may be linked to the species of Symbiodiniaceae with which it associated (Hoogenboom et al., 2017; Putchim et al., 2017; Sakai, Singh & Iguchi, 2019), whereas *P.* cf. *damicornis* containing *Durusdinium* showed fewer effects on the corals in the different seasons. Distinct genera/species of Symbiodiniaceae have been shown to perform differently under changing environments. For example, *Durusdinium* and *Symbiodinium* are considered to be stress-tolerant and/or opportunistic species (ie., warm, high turbidity, high irradiance) (LaJeunesse, 2002; Levas et al., 2013; Rowan, 2004; Wham, Ning & LaJeunesse, 2017) whereas corals associated with *Cladocopium* species exhibited more susceptibility during stressful conditions in some coral reef regions (Suwa, Hirose & Hidaka, 2008; Thinesh et al., 2019).

Our results revealed that the seasonal variation does not affect the dominant genus/species of Symbiodiniaceae in both *A. humilis* and *P.* cf. *damicornis* corals. Most corals have not been shown to change their species or community composition of Symbiodiniaceae, despite continued changes in environmental conditions (Coffroth et al., 2010; Goulet, 2006; Keshavmurthy et al., 2020; Lewis & Coffroth, 2004). There are several studies that have shown a similar trend along tropical to temperate regions (Berkelmans & Van Oppen, 2006; Cunning, Silverstein & Baker, 2018; Rouze et al., 2017). Ng & Ang (2016) who investigated labeled colonies of *Platygyra acuta* and *Porites* spp. from Hong Kong reefs in different seasons found that there was no change in the Symbiodiniaceae species in all corals. Similarly, *Porites lutea* in the Andaman Sea, Thailand, retain a stable Symbiodiniaceae community structure (C116, C15f, C15g, C15 h, C15.2) even in bleached corals (Pootakham et al., 2018). Although, Kagoshima Bay, Japan has a high variability of temperature, there was no variation of Symbiodiniaceae (C1, C3-related) in *Zoanthus saensibaricus* (zoanthids) across twelve months of the experiment (Reimer, 2007). In this way, Symbiodiniaceae shuffling or switching in corals might not be a common mechanism to survive under environmental stress (Goulet, 2006; Keshavmurthy et al., 2020).

Several possible hypotheses could explain why corals maintain a stable community of Symbiodiniaceae during environmental stress; 1) avoiding intraspecific competition among Symbiodiniaceae species that could lead to disequilibrium of nutrients within the coral host and affect coral growth, 2) coral response to historical environment stressors may promote stable Symbiodiniaceae associations, 3) higher composition of some Symbiodiniaceae species may effect coral-cell metabolism, 4) there may be no severe effect on corals after experiencing stressful conditions (Jones & Berkelmans, 2011; Klepac et al., 2015; LaJeunesse et al., 2010; Putnam et al., 2012) or 5) limitation of high-stress tolerant Symbiodiniaceae genotypes within the host coral and/or environment. However, the variation of Symbiodiniaceae diversity and/or communities may be driven by several factors including local environmental variability, coral species background, Symbiodiniaceae supply sources such as seawater, sediment etc. and investigation techniques (Baird et al., 2009; LaJeunesse et al., 2010; Ng & Ang, 2016; Sweet, 2014; Thornhill et al., 2014; Van Oppen et al., 2005). Although the corals-Symbiodiniaceae associations in the upper Gulf of Thailand were found to be stable during the studied year, we predict that increasing severe coral bleaching in the future may result in acclimatization or adaptation of some corals by Symbiodiniaceae shuffling within hosts.

## Conclusions

Both *A. humilis* and *P.* cf. *damicornis* in this study exhibited the lowest zooxanthellae density during the summer, which is likely caused by light intensity. In addition, the corals had a strong relationship with particular Symbiodiniaceae species, specific to each species, and showed no variation of Symbiodiniaceae species throughout the year. The corals in the upper Gulf of Thailand may select the appropriate genus/species of Symbiodiniaceae in response to local environmental stressors. The results of this study provided some information on the coral-Symbiodiniaceae relationship between seasons, which may be applied to predicting the potential adaptation of corals in localized reef environments. However, more coral species and study sites are needed to further study the seasonal variation on coral-Symbiodiniaceae in Thailand.

##  Supplemental Information

10.7717/peerj.13114/supp-1 Supplemental Information 1 Light intensity and temperature during Nov 2017 –Oct 2018

10.7717/peerj.13114/supp-2 Supplemental Information 2 Zooxanthellae density of *Acropora humilis* and *Pocillopra* cf. *damicornis*

10.7717/peerj.13114/supp-3 Supplemental Information 3 Multiple comparisons between zooxanthellae density and seasons

10.7717/peerj.13114/supp-4 Supplemental Information 4 Number of sequencing reads and alpha diversity indices of corals

## Additional Information and Declarations

### DNA Deposition

The following information was supplied regarding the deposition of DNA sequences: The Symbiodiniaceae sequences are available at GenBank: PRJNA747703.

### Data Availability

The following information was supplied regarding data availability: The number of sequencing reads and alpha diversity indicies of two coral species, Acropora humilis and Pocillopora damicornis, is available in the Supplemental Files.
