# Supplementary figures and images for: The seasonal investigation of Symbiodiniaceae in broadcast spawning, Acropora humilis and brooding, Pocillopora cf. damicornis corals
Source: PeerJ. 2022 Jun 14;10:e13114. doi: 10.7717/peerj.13114 (PMC9205303; doi:10.7717/peerj.13114)

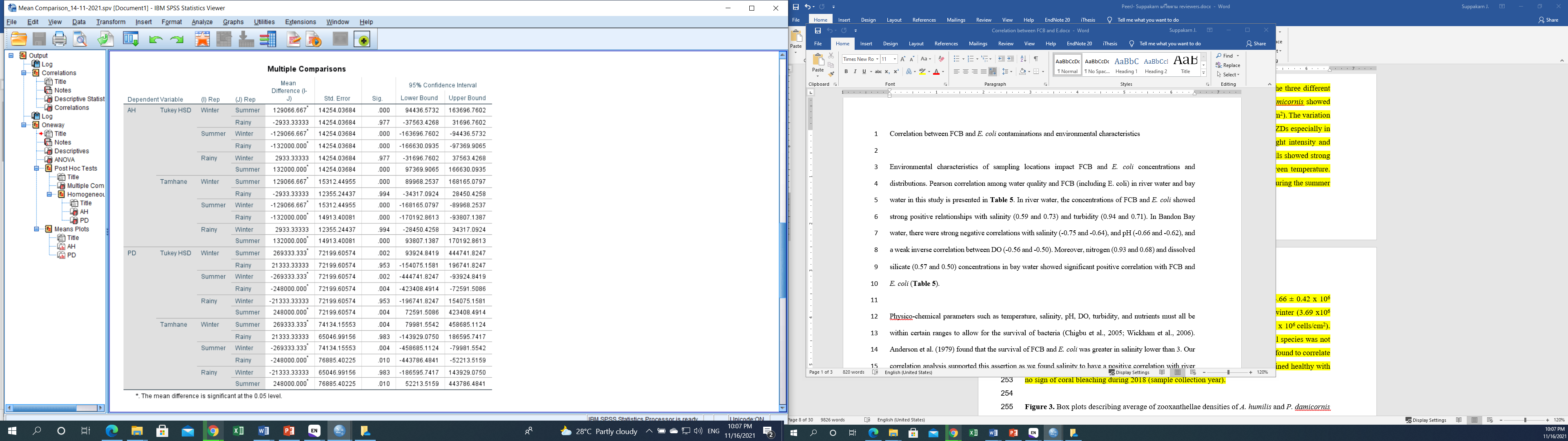

Supplement: Supplemental Information 3 [file peerj-10-13114-s003.docx]
